# The Effect of Analytic Cognitive Style on Credulity

**DOI:** 10.3389/fpsyg.2020.584424

**Published:** 2020-10-15

**Authors:** Eva Ballová Mikušková, Vladimíra Čavojová

**Affiliations:** Institute of Experimental Psychology, Centre of Social and Psychological Sciences, Slovak Academy of Sciences (SAS), Bratislava, Slovakia

**Keywords:** credulity, analytic cognitive style, cognitive reflection, paranormal beliefs, paranormal explanation

## Abstract

Belief in astrology remains strong even today, and one of the explanations why some people endorse paranormal explanations is the individual differences in analytical thinking. Therefore, the main aim of this paper was to determine the effects of priming an analytical or intuitive thinking style on the credulity of participants. In two experiments (*N* = 965), analytic thinking was induced and the source of fake profile (astrological reading vs. psychological testing) was manipulated and participants’ prior paranormal beliefs, anomalous explanation, cognitive reflection, and depression were measured. Although analytic thinking was proved to be hard to induce experimentally, the results showed that analytic thinking predicts credulity and belief in the paranormal was linked with experiencing more anomalous experiences and more paranormal explanations. The more people were able to think analytically, the less credulous they were as reflected in the lower acceptance of fake profile as accurate.

## Introduction

According to the various studies from around the globe, the number of people believing in astrology ranges from 28% (Newport and Strausberg, 2001) to 70.51% (Čavojová and Jurkovič, 2017), depending on the exact question asked and the cultural background of participants. This variability in self-reported belief in astrology depends on whether we ask about personal experience and behavior or objective facts. According to Campion (2017), when researchers ask about personal experience (e.g., “Do you check your partner’s zodiac sign?”), they get almost two times higher ratings than when they ask about objective facts (e.g., “Do you think astrology can make accurate predictions about the future?”). Still, it is unclear why so many people still believe things that have been proven to be untrue time and again. There are several explanations, and the one most accepted refers to individual differences in the propensity to override one’s intuition (De Neys and Bonnefon, 2013) and think analytically (sometimes called also the cognitive reflection), which is rooted in dual-process theories accounts.

Dual-process theories posit two types of processes—one quick, automatic, and relying on intuition and the other slow, deliberate, and dependent on working memory capacity. Relying on intuitive processes can lead to underestimating chance events, attributing mental states to inanimate objects, or misperceiving probability, which are processes contributing to having more paranormal beliefs (Rogers et al., 2009; Irwin et al., 2013; Svedholm and Lindeman, 2013). Analytical processes are necessary to overcome these intuitive processes, but people may fail to notice the necessity to override the intuitive response (detection failure), they may lack necessary knowledge (storage failure), or they may fail to override their intuitive response despite the effort to do so (inhibition failure) (De Neys and Bonnefon, 2013).

Analytic thinking style has been consistently shown to correlate negatively with having epistemically suspect beliefs (Lobato et al., 2014; Čavojová et al., 2020), i.e., beliefs that are not corresponding to reality or not supported by current scientific evidence. Belief in astrology is an example of paranormal beliefs, and it has been shown that people with higher analytic thinking have fewer paranormal beliefs, believe less in conspiracy theories, or are more sensitive to bullshit detection (Pennycook et al., 2015). According to some authors, paranormal beliefs (as well as other epistemically suspect beliefs) are best predicted by intuitive thinking style; however, studies examining the relationship between analytic thinking and more general paranormal beliefs found also a consistent negative association between the analytic thinking and paranormal beliefs, regardless of whether it was measured by cognitive tasks—usually cognitive reflection test (Frederick, 2005)—or self-reported measures (Aarnio and Lindeman, 2005; Lindeman and Aarnio, 2007; Pennycook et al., 2012; Cheyne and Pennycook, 2013; Svedholm, 2013; Svedholm and Lindeman, 2013; Lobato et al., 2014; Čavojová et al., 2018; Ståhl and van Prooijen, 2018; Rizeq et al., 2020). Specifically, lack of cognitive reflection can lead to the acceptance of supernatural causation for experience with astrology and extrasensory perception (ESP), irrespective of one’s prior beliefs in the supernatural, as shown in Bouvet and Bonnefon’s (2015) study. Their findings, related to the acceptance of fake astrology profile, were replicated in a study by Ballová Mikušková and Čavojová (2019).

On the other hand, the majority of these findings come from correlational studies; only few studies so far examined the causal effect of analytic thinking on various kinds of suspicious beliefs, mostly on conspiracy beliefs (Swami et al., 2014) and fake news (Bago et al., 2020; Pennycook et al., 2020). While priming analytic thinking seems to be beneficial and decreases acceptance of conspiracy explanations and fake news, the findings related to the effect of analytical thinking on personal beliefs (e.g., religious beliefs) are inconsistent. For example, while (Gervais and Norenzayan, 2012) found that priming analytical thinking decreased religious beliefs—this result was replicated also in a Turkish sample (Yilmaz et al., 2016)—other studies failed to replicate this result (Shenhav et al., 2012; Deppe et al., 2015; Lutzke et al., 2019). However, despite having some common features like invoking supernatural forces, religious beliefs are different from paranormal beliefs (Aarnio and Lindeman, 2007; Irwin, 2009), and in general, results of various studies suggest that analytic thinking is indispensable in countering epistemically suspect beliefs.

However, despite quite convincing evidence of the role of cognitive reflection and analytic thinking in rejecting acceptation of supernatural causation and epistemically suspect beliefs, there is one alternative explanation for Bouvet and Bonnefon (2015) findings. Did people reject supernatural explanation and distrust the accuracy of fake astrological profiles because they thought about a possible alternative explanation (and the results are driven by their analytic thinking) or they relied on their prior beliefs about astrology (and the results are driven by the cue that astrology is inherently unreliable)? It was already shown that people with higher cognitive reflection or analytic thinking tend to have fewer unfounded beliefs, including a belief in astrology and ESP, and thus their encounter with astrology and ESP could serve as a cue to trigger an analytic process and an alternative explanation in the face of an uncanny event. We were wondering if the participants’ analytic thinking would help them recognize a fake personality profile even if they believed it came from a more reliable source, such as psychology. For this purpose, we used the so-called Barnum profile, which consists of deliberately vague, ambiguous, and general statements describing the personality (Forer, 1949; for a research review, see Dickson and Kelly, 1985). Would participants still recognize the need to engage their deliberate processing or would they believe in the accuracy of the Barnum profile? To tease apart the effect of thinking style (analytic or intuitive) and cue (astrology or psychology), we designed an experimental approach to examine the effect of analytic thinking style on credulity—“the tendency to believe something without critically examining the evidence for that claim” (Greenspan et al., 2001).

Moreover, we expanded the previous research by examining the possible role of negative emotionality on credulity. The decision to examine the effect of negative emotionality on credulity was based on two lines of research. First, it has been shown that negative emotionality or neuroticism increased the paranormal beliefs (Wiseman and Watt, 2004; Mikloušić et al., 2012; Lobato et al., 2014; Betsch et al., 2020) and also that believers are more neurotics than skeptics (Lindeman and Aarnio, 2007). Second, negative emotionality (emotional instability and neuroticism) affects information processing and thus credulity—neuroticism seemed to be a negative predictor of rational processing style (Pacini and Epstein, 1999). Besides the incidental role of emotionality in information processing, there is another line of research focusing on how information processing of depressed individuals differs from people with no signs of clinical depression. In one study, depression was linked to paranormal beliefs and depressive tendencies were associated with ghost-related ideation (Sharps et al., 2006). On the other hand, more relevant to the current study was the suggestion that people with more depressive symptoms have a more realistic view about themselves and others, the so-called depressive realism (Pyszczynski et al., 1987; Alloy and Abramson, 1988). However, one study showed that depressed people tend to be overconfident even more than non-depressed people (Dunning and Story, 1991). Thus, we wanted to examine whether people scoring higher on depression from the Big 5 questionnaire would be better able to see through the ambiguity of a fake profile because depression (1) is linked with more careful processing of information and (2) is linked with a more realistic view of oneself.

In the present paper, we tried to replicate the main effect of analytic cognitive style (cognitive reflection) vs. the effect of source of information (how the profile is “produced”) on credulity (the likelihood to accept supernatural causation). We extend the research (a) by experimental design where we manipulate the analytic cognitive style as well as the source of the profile (in previous research the cognitive reflection was not manipulated) and (b) by controlling prior paranormal beliefs, anomalous experiences, paranormal attribution to these experiences, and depression as possible predictors of credulity. Thus, our main research questions were as follows: Does analytic cognitive style prevent credulity and susceptibility to a paranormal explanation of uncanny events? Or possibly, is it the knowledge of the source of information (astrology vs. psychology) that affects credulity and susceptibility to a paranormal explanation of uncanny events? Further, we expected that prior paranormal beliefs, proneness to anomalous experience and explanation, and depression will predict credulity and susceptibility to paranormal explanations of an uncanny event over and above analytic thinking.

## Study 1

Study 1 was designed to replicate the findings of previous research (Bouvet and Bonnefon, 2015; Ballová Mikušková and Čavojová, 2019) using an experimental design that would allow teasing apart the effect of analytic thinking and the effect of the cue (source of the profile). Specifically, as summarized in the literature review above, we expected:

(H1) If it is the analytic style that prevents credulity, then participants in the analytic condition should perceive their profile as less accurate and should attribute profile accuracy to coincidence regardless of the source of profile (astrology vs. psychology) in comparison to the control group (analytic thinking hypothesis).
(H2) If, on the other hand, it is the information whether the profile was produced by astrology or psychology that cues participants to be more skeptical to fake astrology than fake psychology profile (both being the same), then participants in the astrology condition should perceive their profile as less accurate and should attribute profile accuracy to coincidence, regardless of being in the analytic thinking condition or the control group (cue hypothesis).
(H3) If both analytic cognitive style and source of information (cue) affect credulity, then participants in the analytic thinking condition should perceive their profile as less accurate only when it is produced by astrology and more accurate when produced by psychology.

Moreover, we expect that prior paranormal beliefs predict credulity over and above the effects of manipulation (analytic thinking and source of information) (H4), proneness to anomalous explanation predicts credulity over and above the effects of manipulation (analytic thinking and source of information) (H5), and depression predicts credulity over and above the effects of manipulation (analytic thinking and source of information) (H6).

### Materials and Methods

Study 1 was preregistered^1^ and all analyses are following the preregistered design.^2^

#### Participants

Data were collected through an online survey hosted on Qualtrics, and participants were recruited through an external participant recruitment agency that complies with the ESOMAR international code. The sample size was determined by an a priori power analysis for a two-way ANOVA. The analysis was conducted using an alpha of 0.05, a power of 0.80, and an effect size of 0.25, yielding the sample of 400 + 10%. Data collection was to be terminated after reaching the desired number (440), but the agency involved more participants in the research than planned. The sample consisted of 473 adult people (233 men, 1 did not answer) between the ages of 18 and 67 (*M* = 41.54, *SD* = 13.75), 0.4% with completed elementary education, 73.6% with secondary education, and 26% with tertiary education.

Participants were selected for the sample following predetermined criteria (age 18+, balanced men and women, balanced education, maximum of 10% university students); they were rewarded with points (within the remuneration system of external agency), which can be exchanged for various products. Data were checked by an external agency for unfinished questionnaires, extreme values, time taken to fill in the questionnaire, and peculiar patterns of answers indicating mindless responding (checking all a’s, etc.). To prevent the accidental filling of tasks, we included two control questions (attention checks) such as “If you read this sentence, press 2”. Participants who failed to select the correct answer were excluded from the data set.

The study was carried out following ethical principles introduced by the American Psychological Association. Collected data were only those relevant to the research purpose, and the data were anonymized so they could not be traced. Nor can an individual’s identity be inferred via deductive disclosure.

#### Procedure

We conducted a simple 2 × 2 experiment with a between-participant design. Participants were told that the goal was to study various methods of personality mapping.

First, we measured paranormal beliefs, anomalous experiences, anomalous explanation, and depression. The order of blocks, as well as the order of items in the blocks, were randomized to avoid the order effect. We did not randomize the order of items in the Big Five Inventory-2 (BFI-2; used for measuring depression) because it is set; the item sequence—one item from each dimension—is repeated.

Next, we manipulated their engagement of analytic thinking. Participants were divided into two groups—experimental (analytic thinking condition) and the control group. In the experimental group, we prompted participants to use an analytic cognitive style by the following instruction: “In many life situations, we must think carefully about things. Now, we will test this ability, so when answering the following tasks, please think thoroughly about your response.” In the control group, there was no instruction. Participants then solved six tasks from the Cognitive Reflection Test and answered one self-rating question (which served as a manipulation check).

After the first manipulation (cognitive style vs. control group), participants were randomly divided into two conditions—astrological and psychological. In the astrological condition, participants were asked to provide their date of birth so that their astrological profile could be created. To enhance the esoteric nature of this condition, a mandala appeared on the screen for a few seconds asking participants to wait while their unique astrological profile was being calculated. In the psychology conditions, participants were told that a psychological profile was created based on their score in the previous set of psychological tests. Consequently, we had four groups (sex, age, and education balanced): analytic with an astrology profile (*n* = 118), analytic with a psychological profile (*n* = 121), control with an astrology profile (*n* = 116), and control with a psychological profile (*n* = 118). After manipulation, all participants received the same Barnum description of personality (Forer, 1949; Bouvet and Bonnefon, 2015), which consisted of 12 vague and ambiguous descriptions.

At the end of the survey, we asked participants to write (open question) what they thought the true purpose of the study was. Finally, we explained the process to debrief participants—we explained that all profiles were fake, how they were created, and why deception was necessary to achieve the goals of the study. We provided participants with the authors’ contact in case they had questions or objections.

#### Materials

##### Credulity and randomness

After manipulation, the Barnum profile was presented to all participants. They were asked to rate on a 7-point scale (1 = totally disagree; 7 = totally agree) their agreement with three statements related to perceived accuracy of the profile, two statements related to perceived randomness, one statement related to uncanniness, and one statement related to the accuracy of the method used (the exact wording of items is in Appendix A). A principal component analysis (PCA) was conducted on 7 items for the profile assessment with orthogonal rotation (varimax). Bartlett’s test of sphericity, χ^2^(21) = 1775.59, *p* < 0.001, indicated that correlations between items were sufficiently large for PCA. An initial analysis was run to obtain eigenvalues for each component in the data. Two components had eigenvalues over Kaiser’s criterion of 1 and in combination explained 69.81% of the variance. The scree plot showed inflections that would justify retaining 2 factors. Component 1 represented the credulity (5 items; reliability ω = 0.887; α = 0.874), and Component 2 represented paranormal attribution–paranormal explanation of what happened (2 items; reliability ω = 0.623; α = 0.623). The score for credulity was computed as the mean score of profile and procedure accuracy items, and the score for paranormal attribution was computed as the mean score of uncanniness items; a higher score indicated higher credulity and paranormal attribution.

##### Manipulation check

As a manipulation check, we asked participants to rate how thoroughly they thought about their answers in CRT on a 7-point scale (1 = I didn’t think of my answers at all; 7 = I have carefully considered my answers); the higher score in self-rating question indicated that participants paid attention to our instruction.

##### Analytic cognitive style

We asked participants to solve six tasks of a modified version of the CRT (Frederick, 2005; Sirota et al., 2019)—three mathematical (e.g., “A pencil and an eraser cost 1.10€ in total. The eraser costs 1.00€ more than the pencil. How much does the pencil cost?”) and three verbal tasks (e.g., “If you’re running a race and you pass the person in second place, what place are you in?”)—in which participants have to override their initial intuitive (and incorrect) response to come to the correct solution. The number of correct responses served as a measure of analytic cognitive style–cognitive reflection. We expected that analytic thinking prompt would increase participants’ awareness about the tricky nature of this test—the higher score did indicate a higher analytic cognitive style.

##### Paranormal beliefs

We used the revised 26-item Paranormal Belief Scale (Tobacyk, 2004)—a measure of the self-reported degree of belief in paranormal phenomena—measuring witchcraft (4 items, e.g., “Black magic really exists.”), superstition (3 items, e.g., “Black cats can bring bad luck.”), spiritualism [4 items, e.g., “Your mind or soul can leave your body and travel (astral projection).”], psi (4 items), and precognition (4 items, e.g., “Some psychics can accurately predict the future.”); religious beliefs (4 items, e.g., “There is a devil.”); and extraordinary life forms (3 items, e.g., “The Loch Ness monster of Scotland exists.”). Two items from precognition regarding astrology were excluded from the analysis. Participants rated items on a 7-point scale (1 = totally disagree; 7 = totally agree). A higher score indicated higher prior paranormal beliefs.

##### Anomalous experience and explanation

The Survey of Anomalous Experience (SAE; Irwin et al., 2013) comprises 20 items addressing anomalous experiences (e.g., “With someone I know intimately I sometimes know what they are about to say before they say it.”). Participants have to indicate whether something like that has already happened to them and further clarify their position by stating whether they attributed this experience to a paranormal process (e.g., “Yes, and it must have been an instance of telepathy or ESP.”) or to a specified non-paranormal process (e.g., “Yes, but it was probably just a lucky guess based on my familiarity with that particular person.”). Based on the answers, two scores were computed: proneness to anomalous experiences was the percentage of the “yes” answers (option 1 or 2) and proneness to an anomalous explanation was the percentage of supernatural attribution to these anomalous experiences (percentage of the “yes, paranormal” answers).

##### Depression

To measure depression, we used The Big Five Inventory–2 (BFI-2; Halama et al., 2020), and participants rated on a 5-point scale (1 = totally disagree; 5 = totally agree) the extent to which 60 statements describe them. We computed a mean score from 4 items (e.g., “I’m someone who often feels sad.”) measuring depression, a higher mean score indicates a higher depression.

##### Demographic characteristics

We asked participants to indicate their age, gender, and their experience with astrology. Participants had to answer a multiple-choice question to indicate which astrological experience relates to them (the options were: “I have no experience with astrology.”; “No personal experience, but persons close to me believe in astrology.”; “Sometimes I look at a horoscope.”; “I read my horoscope and I follow it.”; and “I know how to develop an astrological profile for myself or others.”). A higher score indicated a higher experience with astrology.

### Results and Discussion

The descriptive statistics of all measured variables are in Table 1. First, age and gender differences among groups were tested—we found neither age [*F*(3,201) = 0.598, *p* = 0.617] nor gender [χ^2^(205) = 2.976; *p* = 0.395] differences among groups. Second, we controlled whether analytic cognitive thinking manipulation worked—we compared (Student’s *t*-test) the experimental and control groups in their score of self-rating of deliberate thinking engagement. We found that manipulation did not work—the score in the experimental group was higher than in the control group, but the difference was not significant (*t* = 1.319; *p* = 0.188).

**TABLE 1:** Descriptive statistics and correlations for measured variables in Study 1.

	ω	*M*	*SD*	1	2	3	4	5	6	7
(1) Credulity	0.887	4.18	1.42	–						
(2) Paranormal attribution	0.623	3.54	1.48	0.111*	–					
(3) Manipulation check	–	5.27	1.30	0.150**	0.122**	–				
(4) CRT	0.663	2.05	1.66	−0.249**	0.009	0.121*	–			
(5) PBS (without astro-items)	0.925	3.52	1.16	0.334**	−0.123**	0.074	−0.149**	–		
(6) Anomalous experience	0.717	71.36	16.58	−0.111*	−0.128**	–0.047	–0.047	0.036	–	
(7) Anomalous explanation	0.885	19.18	24.50	0.270**	−0.117*	0.083	−0.144**	0.469**	0.165**	–
(8) BFI-2 depression	0.766	2.77	0.84	0.064	0.057	–0.066	–0.049	0.071	−0.094*	0.038

N = 473. The table shows the mean score (M), standard deviations (SD), and internal consistency (ω) of the measures reported in the study. Significance: *p < 0.05, **p < 0.01, ***p < 0.01.

Because manipulation did not work, hierarchical multiple regression was conducted separately for credulity and paranormal attribution as the dependent variables. The source of information (dummy variable, psycho = 0, astro = 1) was entered in the first step, analytic cognitive style (CRT) in the second step, prior paranormal beliefs (PBS), and proneness to anomalous explanation (PAE) in the third step of regression (depression did not correlate with credulity or paranormal attribution, so it was not entered in regression). Regression statistics are in Tables 2A,B, separately for credulity and paranormal attribution.

**TABLE 2:** Summary of hierarchical regression analysis for variables predicting credulity in Study 1.

Predictors	*B*	*SE*	β	*t*	*p*	95% CI
1 (Constant)	4.261	0.092		46.344	<0.001	[4.1, 4.4]
Psycho = 0, astro = 1	–0.159	0.131	–0.056	–1.219	0.223	[−0.4, 0.1]
	
	*F*(1,471) = 1.486, *p* = 0.223, *R*^2^ = 0.003					

2 (Constant)	4.711	0.120		39.338		[4.5, 4.9]
Psycho = 0, astro = 1	–0.179	0.127	−0.063	–1.414	0.158	[−0.4, 0.1]
CRT	–0.215	0.038	−0.251	–5.626	<0.001	[−0.3, −0.1]
	
	*F*(2,470) = 16.616, *p* < 0.001, *R*^2^ = 0.066, *R*^2^Δ = 0.063					

3 (Constant)	3.418	0.226		15.141	<0.001	[3.0, 3.9]
Psycho = 0, astro = 1	–0.217	0.120	–0.076	–1.810	0.071	[−0.5, 0.0]
CRT	–0.168	0.037	–0.196	–4.586	<0.001	[−0.2, −0.1]
PBS	0.350	0.059	0.249	5.206	<0.001	[0.2, 0.4]
PAE	0.007	0.003	0.127	2.649	0.008	[0.0, 0.01]
	*F*(4,468) = 24.074, *p* < 0.001, *R*^2^ = 0.171, *R*^2^Δ = 0.105					

**TABLE 7:** Summary of hierarchical regression analysis for variables predicting paranormal attribution in Study 1.

Predictors	*B*	*SE*	β	*t*	*p*	95% CI
1 (Constant)	3.603	0.096		37.670	<0.001	[3.4, 3.8]
Psycho = 0, astro = 1	–0.132	0.136	–0.045	–0.974	0.331	[−0.4, 0.1]
	
	*F*(1,471) = 0.949, *p* = 0.331, *R*^2^ = 0.002					

2 (Constant)	3.622	0.129		28.148	<0.001	[3.4, 3.9]
Psycho = 0, astro = 1	–0.133	0.136	–0.045	–0.979	0.328	[−0.4, 0.1]
CRT	–0.009	0.041	–0.011	–0.230	0.818	[−0.1, 0.1]
	
	*F*(2,470) = 0.500, *p* = 0.607, *R*^2^ = 0.002, *R*^2^Δ = 0.000					

3 (Constant)	3.090	0.255		12.127	<0.001	[2.6, 3.6]
Psycho = 0, astro = 1	–0.150	0.135	–0.051	–1.108	0.268	[−0.4, 0.1]
CRT	0.012	0.041	0.014	0.300	0.764	[−0.1, 0.1]
PBS	0.115	0.066	0.091	1.741	0.082	[−0.02, 0.2]
PAE	0.005	0.003	0.078	1.504	0.133	[0.0, 0.01]
	*F*(4,468) = 2.682, *p* = 0.031, *R*^2^ = 0.022, *R*^2^Δ = 0.020					

#### Credulity

The hierarchical multiple regression revealed that at step 1, the source of information did not contribute significantly to the regression model. Introducing the analytic cognitive style (CRT) explained an additional 6.3% of the variation in credulity, and this change in *R*^2^ was significant, *F*(1,470) = 16.616, *p* < 0.001. Finally, introducing paranormal beliefs (PBS) and anomalous explanation (PAE) explained an additional 10.5% of the variation in credulity, and this change in *R*^2^ was significant, *F*(4,468) = 24.074, *p* < 0.001.

#### Paranormal Attribution

The hierarchical multiple regression revealed that at step 1, the source of information did not contribute significantly to the regression model. Neither did the introduction of analytic cognitive style (CRT) in step 2. Introducing paranormal beliefs (PBS) and anomalous explanation (PAE) explained an additional 2.0% of the variation in credulity, and this change in *R*^2^ was significant, *F*(4,468) = 2.682, *p* < 0.031, but there was no significant predictor of the paranormal attribution.

The aim of study 1 was to verify the findings of previous research (Bouvet and Bonnefon, 2015; Ballová Mikušková and Čavojová, 2019), namely, that analytic thinking is the main cause behind skepticism toward fake profile, regardless of whether the fake profile is supposedly produced by astrology or more “mundane” psychology. Although we were unable to experimentally induce analytic thinking, similarly to previous studies, we did find that analytic thinkers did not fall for the fake profile as much as non-analytic thinkers. Moreover, the effect of the source of profile (whether the profile was produced by astrology or psychology) was not significant, and, in contrast with Bouvet and Bonnefon (2015, Study 1), we found that credulity was significantly associated with paranormal attribution and predicted by previous paranormal beliefs and paranormal explanation of uncanny events. Our findings suggest that people in the psychology condition were as credulous as people in the astrology condition and that people who in general accepted more paranormal phenomena and paranormal explanations as true found fake profiles as more believable. Bouvet and Bonnefon (2015) also found the effect of prior paranormal beliefs in their Studies 2 and 3, in which they did not examine uncanny experience with astrology but with extrasensory perception. It seems reasonable to assume that the difference was caused by smaller sample size in their study, and the results altogether indicate that prior beliefs possibly play at least some role in the way how people interpret new uncanny experience.

The main limit of this study, however, was that we were not successful in the manipulation of analytic thinking and thus were not able to establish the causal role analytic thinking may play in explaining uncanny events in a non-supernatural way. Based on the dual-process theory, in addition to analytic thinking people use also intuitive thinking—thinking that is associated with fast, automatic, and default processes and with cognitive miserliness (e.g., Pacini and Epstein, 1999; Stanovich and West, 2000; Evans, 2003). Moreover, intuitive thinking is related to superstitions and paranormal beliefs and is considered as a source from which superstitions arise (Epstein et al., 1996; Aarnio and Lindeman, 2005; Lindeman and Aarnio, 2007). These suggest that people, who rely on intuition could be more credulous—more prone to see the meaning in ambiguous stimuli and, therefore, may also be more prone to accept fake personality profiles; it is possible that intuitive thinking could be a stronger predictor of credulity. Therefore, we decided to replicate the Study 1, the only difference being stronger manipulation of analytic thinking and also inducing intuitive thinking.

## Study 2

Study 2 aimed to provide a direct replication of Study 1, with two modifications. Because in Study 1 manipulation of analytic thinking through simple instruction did not work, in Study 2 we extended the manipulation form (instruction) through the task inducing analytic cognitive style. The second modification was adding another experimental condition, in which we induced intuitive cognitive style—similarly through instruction and task inducing intuitive cognitive style. Several studies have shown a positive relationship between intuitive thinking (faith in intuition) and superstitions, and this relationship seemed to be stronger than an association of analytical thinking and superstitions (Epstein et al., 1996; Aarnio and Lindeman, 2005; Lindeman and Aarnio, 2007). Lindeman (2018; p. 41) summed up that “…people who believe in supernatural phenomena … prefer to follow their instincts and rely on their intuition.” So, in Study 2 we examined whether intuitive thinking would be a stronger predictor of credulity than analytic thinking.

### Materials and Methods

Study 2 was preregistered,^3^ and all analyses are following preregistered design.^4^

#### Participants

Similarly, as in Study 1, data were collected by the same participant recruitment agency as in Study 1, but the agency distributed our online survey hosted on Qualtrics to different participants. The analysis was conducted using an alpha of 0.05, a power of 0.95, an effect size of 0.25, and the number of groups 6. Based on the aforementioned assumptions, the desired sample is 400 + 10%. Data collection was to be terminated after reaching the desired number (440); participant recruitment agency involved more participants in the research as planned. The final sample consisted of 492 adult people (230 men, 1 did not answer) between the ages of 18 and 66 (*M* = 39.79, *SD* = 13.68): 3.7% with completed elementary education, 73.0% with secondary education, and 23.3% with tertiary education.

#### Procedure

We conducted a simple 3 × 2 experiment with between-participant design. Participants were told that the goal was to study various methods of personality mapping. The design was the same as in Study 1, only intuitive thinking condition was added, and manipulation was strengthened.

In the analytic experimental group, we prompted participants to use analytic cognitive style by the following instruction: “In many life situations, we must think carefully about things. Now, we will test this ability, so when answering the following tasks, please think thoroughly about your response.” A task designed to further induce analytic thinking (taken from Gervais and Norenzayan, 2012) followed: participants received 5 sets of five randomly arranged analytic thinking words (e.g., mark, justify, my, detail, and decision) and had to drop one word and make a meaningful sentence out of the rest of the words. This method was chosen mainly because it represented a deliberate effort to reduce potential effects of experimental demand (Gervais and Norenzayan, 2012)—it was easy to use in an online setting, and it used subtle techniques to induce analytical thinking. Moreover, the authors claimed that participants only rarely detected a connection between manipulations and their variables of interest, which was important for our design, as well.

In the intuitive experimental group, we supported the intuitive cognitive style with the following instruction: “In many life situations, we should decide fast, following our first inclination and gut feelings. Now, we will test this ability, so when answering the following tasks, please respond quickly and listen to your gut feelings.” and with a task inducing intuitive cognitive style. Since the task inducing intuitive cognitive style was inspired by the study of (Oberman and Ramachandran, 2008), participants had to pair nonsense shapes with nonsense words (shapes were presented in pairs with a pair of words).

In the control group, there was no instruction. Then, participants solved six tasks from the Cognitive Reflection Test as a measure of analytic thinking and answered one self-rating question on how thoroughly they thought about the answers as a manipulation check.

After the first manipulation (analytic and intuitive cognitive style vs. control group), participants were divided into two conditions—astrological and psychological as in Study 1. Finally, we had six groups (sex, age, and education balanced): analytic experimental with astrology profile (*n* = 80) and with psychological profile (*n* = 84), intuitive experimental with astrology profile (*n* = 82) and with psychological profile (*n* = 84), and control with astrology profile (*n* = 80), and with psychological profile (*n* = 82). After this manipulation, all participants received the same Barnum description of personality used by Forer (1949) and Bouvet and Bonnefon (2015), which consists of 12 vague and ambiguous descriptions.

At the end of the survey, we asked participants to write (open question) what they thought the true purpose of the study was. Finally, we explained the process to debrief participants—we explained that all profiles were fake, how they were created, and why deception was necessary to achieve the goals of the study. We provided participants with the authors’ contact in case they had questions or objections.

#### Materials

In Study 2, we used the same methods as in Study 1 to measure credulity and paranormal explanation (assessment of Barnum’s profile), manipulation check (self-rating question), analytic cognitive style (CRT), paranormal beliefs (PBC^5^), anomalous experience, and explanation (SAE), and depression (BFI-2). Descriptive statistics and correlations for all measured variables are in Table 3.

**TABLE 3:** Descriptive statistics and correlations for measured variables in Study 2.

	ω	*M*	*SD*	1	2	3	4	5	6	7
(1) Credulity	0.870	4.17	1.33	–						
(2) Paranormal attribution	0.597	3.59	1.39	0.190*	–					
(3) Manipulation check	−	5.10	1.83	0.067	−0.105*	–				
(4) CRT	0.706	2.14	1.75	−0.204**	–0.005	0.163**	–			
(5) PBS (without astro-items)	0.929	3.51	1.41	0.338**	0.161*	–0.032	−0.066	–		
(6) Anomalous experience	0.885	0.42	0.23	0.250**	0.055	−0.104*	−0.074	0.493**	–	
(7) Anomalous explanation	0.739	0.25	0.30	0.258**	0.167**	–0.082	−0.064	0.525**	0.464**	–
(8) BFI-2 depression	0.641	2.81	0.78	0.142**	0.001	–0.064	−0.065	0.077	0.073	0.043

N = 492. The Table 3 shows the score (M), standard deviations (SD), and internal consistency (ω) of the measures reported in the study. Significance: *p < 0.05, **p < 0.01, ***p < 0.01.

### Results and Discussion

The descriptive statistics of all measured variables are in Table 3. There were no age [*F*(5,486) = 0.278; *p* = 0.925] or gender [χ^2^(492) = 8.906; *p* = 0.541] differences among groups.

Next, we checked whether cognitive thinking manipulation worked—we compared experimental (analytic and intuitive) and control group (1-way ANOVA) in their score of self-rating of deliberate thinking engagement (manipulation check). There were significant differences among groups [*F*(2,489) = 4.505; *p* = 0.012]. The score in the analytic experimental group (*M* = 5.35, *SD* = 1.69) was significantly higher than in the intuitive group (*M* = 4.77, *SD* = 1.93; *p* = 0.039). There were no significant differences between the analytic and control groups, but participants in the intuitive group reported significantly lower deliberate thinking engagement than those in the control group (*M* = 5.19, *SD* = 1.82; *p* = 0.004). Thus, it seems that while manipulation of intuitive thinking was successful, even the more pronounced manipulation of analytic thinking in comparison with Study 1 did not have the required effect on increasing analytical thinking.

Two-way ANOVA was used for verification of the effect of thinking mode, source of information, and their interaction on the credulity score. We found no main effect of thinking mode (*F* = 0.166, *p* = 0.847), no main effect of source of information (*F* = 0.075, *p* = 0.784), nor their interaction (*F* = 0.860, *p* = 0.424). Because none of our manipulations had the intended effect, we proceeded to analyze what other variables predicted credulity and explanation.

To verify H4–H6, we used a hierarchical multiple regression with the source of information in the first step (dummy variable, psycho = 0, astro = 1); analytical thinking (CRT) which entered in the second step; and prior paranormal beliefs (PBS), proneness to anomalous explanation (PAE), and depression (only for credulity) which entered in the third step which was conducted separately for credulity and paranormal attribution as the dependent variables (Tables 4A,B).

**TABLE 4:** Summary of hierarchical regression analysis for variables predicting credulity in Study 2.

Predictors	*B*	*SE*	β	*t*	*p*	95% CI
1 (Constant)	4.117	0191		21.277	<0.001	[3.7, 4.5]
Psycho = 0, astro = 1	0.033	0.120	0.012	0.273	0.785	[−0.2, 0.3]
	
	*F*(1,491) = 0.075, *p* = 0.785, *R*^2^ = 0.000					

2 (Constant)	4.466	0.202		22.131		[4.1, 4.9]
Psycho = 0, astro = 1	0.021	0.118	0.008	0.176	0.860	[−0.2, 0.3]
CRT	–0.155	0.034	–0.204	–4.603	<0.001	[−0.2, −0.1]
	
	*F*(2,489) = 10.663, *p* < 0.001, *R*^2^ = 9.042, *R*^2^Δ = 0.038					

3 (Constant)	2.879	0.307		9.381	<0.001	[2.3, 3.6]
Psycho = 0, astro = 1	0.050	0.110	0.019	0.456	0.649	[−0.2, 0.3]
CRT	–0.131	0.032	–0.173	–4.148	<0.001	[−0.2, −0.1]
PBS	0.250	0.046	0.266	5.442	<0.001	[0.2, 0.3]
PAE	0.454	0.214	0.103	2.118	0.035	[0.0, 0.9]
BFI-2 depression	0.178	0.071	0.105	2.523	0.012	[0.0, 0.3]
	*F*(5,486) = 19.462, *p* < 0.001, *R*^2^ = 0.167, *R*^2^Δ = 0.158					

**TABLE 8:** Summary of hierarchical regression analysis for variables predicting paranormal attribution in Study 2.

Predictors	*B*	*SE*	β	*t*	*p*	95% CI
1 (Constant)	3.207	0.199		16.101	<0.001	[2.8, 3.6]
Psycho = 0, astro = 1	0.253	0.125	0.091	2.020	0.044	[0.0, 0.5]
	
	*F*(1,491) = 4.080, *p* = 0.044, *R*^2^ = 0.008					

2 (Constant)	3.303	0.215		15.369	<0.001	[2.9, 3.7]
Psycho = 0, astro = 1	0.250	0.125	0.090	1.994	0.047	[0.0, 0.5]
CRT	–.042	0.036	–0.053	–1.182	0.238	[−0.1, −0.1]
	
	*F*(2,489) = 2.740, *p* < 0.066, *R*^2^ = 0.011, *R*^2^Δ = 0.007					

3 (Constant)	2.765	0.272		10.164	<0.001	[2.2, 3.3]
Psycho = 0, astro = 1	0.268	0.124	0.096	2.167	0.031	[0.2, 0.5]
CRT	–0.031	0.035	–0.039	–0.880	0.379	[−0.1, 0.0]
PBS	0.103	0.051	0.104	1.996	0.046	[0.0, 0.2]
PAE	–0.514	0.240	0.111	2.139	0.033	[0.0, 1.0]
	*F*(5,486) = 5.896, *p* < 0.001, *R*^2^ = 0.046, *R*^2^Δ = 0.038					

#### Credulity

The hierarchical multiple regression revealed that at step 1, the source of information did not contribute significantly to the regression model. Introducing the analytic cognitive style (CRT) explained an additional 3.8% of variation in credulity, and this change in *R*^2^ was significant, *F*(2,489) = 10.633, *p* < 0.001. Finally, introducing paranormal beliefs (PBS), anomalous explanation (PAE), and depression explained an additional 15.8% of the variation in credulity, and this change in *R*^2^ was significant, *F*(5,486) = 19.462, *p* < 0.001. Analytic thinking, prior paranormal beliefs, paranormal explanation, and depression significantly predicted credulity—more depressed people and people lower in analytic thinking, with more paranormal beliefs, and with paranormal explanations were more credulous.

#### Paranormal Attribution

The hierarchical multiple regression revealed that at step 1, the source of information contributed significantly to the regression model: the source of information explained 0.8% of the variation in paranormal attribution, and this change in *R*^2^ was significant, *F*(1,490) = 4.080, *p* = 0.044. The introducing analytic cognitive style (CRT) did not explain any additional variation in randomness. Finally, introducing paranormal beliefs (PBS), anomalous explanation (PAE), anxiety, and depression explained an additional 3.8% of the variation in randomness and this change in *R*^2^ was significant, *F*(4,486) = 5.896; *p* < 0.001. The source of information, prior paranormal beliefs, and paranormal explanation significantly predicted paranormal attribution—people in astrology condition, with more paranormal beliefs and paranormal explanations, tended to attribute more paranormal explanation of the profile.

The aim of Study 2 was to replicate the results of Study 1. The main finding was that analytic thinking and prior paranormal beliefs, as well as depression, predicted credulity. These results are in line with our Study 1 and previous studies (Bouvet and Bonnefon, 2015; Ballová Mikušková and Čavojová, 2019) with one exception—our findings are not in line with the depressive realism hypotheses (Pyszczynski et al., 1987; Alloy and Abramson, 1988). On the contrary, we found depression (as a trait) a positive predictor of credulity.

In contrast with Study 1, the source of the profile (astrology vs. psychology) was a significant predictor of perceived paranormal attribution, even after cognitive reflection, paranormal beliefs, and paranormal explanation were introduced into the model. People in the astrology condition tended to see the accuracy of the profile as evidence that astrology works and not as a result of random coincidence or lucky chance. Cognitive reflection did not show as a significant predictor; again prior paranormal beliefs and tendency to anomalous explanations both predicted that people would attribute the accuracy of the profile less to the random explanation and more as evidence that astrology works.

## General Discussion

We aimed to replicate the main effect of analytic cognitive style on the likelihood to accept supernatural causation vs. the effect of source of information (whether the personality profile is “product” of psychology vs. astrology)—we wanted to distinguish between the effect of analytic thinking and relying on cues about the suspicious source of information (astrology). We did, indeed, verify that analytic thinking predicts credulity.

First, the more people were able to think analytically (to postpone judgment and verify their intuitions), the less credulous they were as reflected in the lower acceptance of fake profiles as accurate. Generally, our results extend the findings of Bouvet and Bonnefon (2015): people’s abilities and beliefs they already possess have a stronger effect than the contextual effects, such as encouraging people to think more deliberately and carefully or manipulating the way they receive information (more reliable vs. unreliable source of the fake profile). Both in Study 1 and Study 2, non-reflective thinkers tended to view fake profiles as more accurate (thus were more credulous) regardless of how the profile was produced, while the opposite was true for more reflective thinkers. This finding is consistent with several recent studies that found that analytical thinking is crucial when distinguishing fake news from real news regardless of whether the stories are consistent or inconsistent with one’s political ideology (Pennycook and Rand, 2019; Bago et al., 2020).

Second, in our study, belief in the paranormal was linked with experiencing more anomalous experiences and more paranormal explanations. It indirectly corroborates the assumption that beliefs shape how we perceive what we experience. It is not probably the case that believers are believers because they encountered uncanny events more often but that they experience events as uncanny and supernatural precisely because they are believers and thus tend to see more supernatural phenomena where other people see either random events or do not notice anything uncanny at all. In the study of Irwin and Wilson (2013), both a proneness to anomalous experiences and a proneness to attribute these experiences to paranormal factors were predicted by an intuitive thinking style, but not by a rational thinking style. Similarly, Schienle et al. (1996) found that strong prior beliefs tended to influence the person’s judgment about the covariation of observed events in line with a prior conviction, even if situational information was incongruent with the belief. They examined this effect in believers of extra-sensory perception (also an instance of paranormal belief) and found that the biased perception (overestimation of correct “telepathic transmission”) was partly mediated by physiological arousal.

Similarly, as in previous studies (Gervais and Norenzayan, 2012; Pennycook et al., 2012; Shenhav et al., 2012; Razmyar and Reeve, 2013), believers in the paranormal had a lower cognitive reflection, but this was true only in Study 1; in Study 2, there was no correlation between paranormal beliefs and cognitive reflection. Similarly, while we found a negative correlation between cognitive reflection and anomalous explanation in Study 1, there was no correlation between the two in Study 2. A possible explanation is that to “unbelieve” in the supernatural requires cognitive effort and only people more prone to analytic thinking are able (and willing enough) to see the inconsistencies between natural and supernatural (Pennycook et al., 2012, 2014).

Finally, we did not find strong support for the link between depression and higher credulity. In Study 1, credulity did not correlate with depression (as a personal trait); in Study 2, the relationship between credulity and depression was weak (*r* = 0.142). This might suggest that depression could be associated with credulity, but a more detailed examination is needed. Two things should be noted on the present research; first, we worked with non-clinic samples, so the depression was on medium level (2.77–2.81 from 5 points). Second, we focused on credulity as reflected by pronounced belief in the accuracy of astrological profiles, which is quite prevalent, and thus the relationship between depression and other specific paranormal beliefs might differ. Moreover, research also suggests that positive emotions (happiness and surprise) lead people to be more accurate in decision making and search for information for a longer time in comparison with negative emotions, such as anger and fear (Chuang, 2007). Moreover, emotions can have a moderating effect through the degree of their activation (Valiente et al., 2012)—even too much of a positive emotional state can decrease the attention and thus affect the whole performance.

There may be a few possible explanations of not quite successfully distinguishing between the effect of analytic thinking vs. the effect of the cue. Analytic thinking proved to be hard to induce experimentally, and the failure to replicate the priming effects on analytical thinking was reported also by other studies (Deppe et al., 2015; Yonker et al., 2016; Sanchez et al., 2017). In Study 2, people in experimental analytic conditions did report that they engaged in more deliberate thinking in comparison with people in experimental intuitive condition; however, increased effortful thinking was reflected neither in the results of our experiment nor in their score of cognitive reflection. These results corroborate findings that people’s perceived skills and their actual skills often do not correlate (Kruger and Dunning, 1999; Dunning, 2005)—although people think that they put more effort into thinking, they do not think more deeply or effectively. Of course, not all participants might perceive psychology as a sufficiently reliable source of information. On the other hand, it is also possible that priming the analytic thinking is not effective enough to influence acceptance of epistemically suspect beliefs, even though these beliefs are affected by the cognitive style. First of all—as shown also by the results in our Study 2—it may be actually easier to prime intuitive thinking style because it does not require any active effort from the participants—just to fall back on their automatic intuitive processes. Priming analytical thinking is dependent on the underlying actual cognitive abilities of participants. We may have succeeded in increasing the effort of people to engage in deliberate processing, but they still failed on inhibiting the intuitive response [inhibition failure (De Neys and Franssens, 2009; De Neys and Bonnefon, 2013)]. This explanation is also supported by studies that found correlations between analytic thinking and various personal beliefs, even though the priming analytic thinking did not affect the scores in the cognitive reflection test (Deppe et al., 2015). It is also possible that the direction of the relationship between paranormal beliefs and analytic thinking goes in the opposite direction—that non-reflective thinkers have less tolerance for uncertainty than non-reflective thinkers that can lead to an endorsement of more supernatural explanations than to accepting chance as a possible explanation.

## Conclusion

In our research, we attempted to show the causal role of analytic thinking in credulity and to experimentally verify the role of the source of information (reliable vs. unreliable). Although we were not successful in manipulating cognitive style and thus reliably distinguishing between the effect of analytic thinking vs. the effect of the cue (astrology as an unreliable source of information), we brought new and robust evidence that more reflective thinkers have fewer paranormal beliefs, use less paranormal explanation after the encounter of uncanny experiences, and are generally less accepting of vague and ambivalent descriptions of personality, which makes them more resistant toward scams and frauds. More reflective people have a long history of questioning suspicious sources of information, and even though they might rely on situational cues at times (such as perceiving astrology as an unreliable source of information and not giving more thought to evaluating the profile), these cues are usually valid indicators.

To return to the question at the beginning—why so many people believe things such as astrology—a glimpse of an answer is provided by the lack of cognitive reflection. The good part is that analytic thinking does, indeed, help to see through the attempts to fool people. The bad part is that it is easy to think we use analytic thinking even when we do not.

## Data Availability Statement

The datasets generated for this study can be found in online repositories. The names of the repository/repositories and accession number(s) can be found below: OSF 10.17605/OSF.IO/WQYSP.

## Ethics Statement

Ethical review and approval was not required for the study on human participants in accordance with the local legislation and institutional requirements. The patients/participants provided their written informed consent to participate in this study and were fully debriefed after termination of the study.

## Author Contributions

EBM and VČ conceived of the presented idea. VČ developed the theory and performed the computations. EBM performed the computations. Both authors discussed the results and contributed to the final manuscript.

## Conflict of Interest

The authors declare that the research was conducted in the absence of any commercial or financial relationships that could be construed as a potential conflict of interest.

## Footnotes

1 https://osf.io/u5pzc/?view_only=e79a5f42cf8442aba88fe83d1cc92cc7

2 Being notified by the reviewers, we made some changes in Study 1 and Study 2:(1) We realized that what we actually measured was credulity not gullibility as we proposed in pre-registration, so we have changed the terminology.(2) The score of paranormal beliefs (measured by Paranormal Belief Scale) was computed without two astrology/horoscope items to avoid using a measure that is known to be correlated with belief in astrology and was designed to be so.

3 https://osf.io/zj32f/?view_only=49f0f513d132404c859cb40a02610ae9

4 We did the same changes in the Study 2 as in the Study1:(1) We changed the term gullibility to the term credulity.(2) The score of paranormal beliefs (measured by PBS) was computed without two astrology/horoscope items.

5 In study 2, we used a 17-item PBS with items measuring witchcraft (4 items), spiritualism (4 items), psi (4 items), precognition (4 items), and traditional religious beliefs (2 items); we excluded items measuring superstition, extraordinary life forms, and some traditional religious beliefs.
