# Corrigendum: The Effect of Analytic Cognitive Style on Credulity

**DOI:** 10.3389/fpsyg.2021.719330

**Published:** 2021-06-24

**Authors:** Eva Ballová Mikušková, Vladimíra Čavojová

**Affiliations:** Institute of Experimental Psychology, Centre of Social and Psychological Sciences, Slovak Academy of Sciences (SAS), Bratislava, Slovakia

**Keywords:** credulity, analytic cognitive style, cognitive reflection, paranormal beliefs, paranormal explanation

In the original article, the reference for Betsch et al., 2020 was incorrectly written as “Betsch, T., Leonie, A., and Glockner, A. (2020). Paranormal beliefs and individual differences: story seeking without reasoned review. *Heliyon* 6, 1–8. doi: 10.1016/j.heliyon.2020.e04259.”

It should be “Betsch, T., Aßmann, L., and Glockner, A. (2020). Paranormal beliefs and individual differences: story seeking without reasoned review. *Heliyon* 6, 1–8. doi: 10.1016/j.heliyon.2020.e04259.”

The authors apologize for this error and state that this does not change the scientific conclusions of the article in any way. The original article has been updated.
