# Supplementary material for: The Effect of Analytic Cognitive Style on Credulity
Source: Front Psychol. 2020 Oct 15;11:584424. doi: 10.3389/fpsyg.2020.584424 (PMC7593255; doi:10.3389/fpsyg.2020.584424)
Supplement: Supplementary file 1 [file Data_Sheet_1.docx]

Supplementary Material

# Appendix A

**The assessment of the Barnum profile**

7-point scale (1= disagree; 7= agree)

1) the profile perfectly describes me

2) the profile is unique for me

3) the profile completely reveals my personality

4) the profile is the result of chance, coincidence, happiness (R)

5) these statements can characterize many people, regardless of the date of birth (R)

6) the profile is the result of something uncanny, mysterious

7) this way of creating a profile is very accurate
